# Predictive factors for mortality in acute mesenteric ischemia

**DOI:** 10.3389/fsurg.2026.1805697

**Published:** 2026-04-10

**Authors:** Yu Chen, Chen Wang, Yuqiang Tang, Ming Ma, Qianhui Ma, Zhengdong Zhao, Ting Liu, Fuwen Luo

**Affiliations:** 1 Department of General Surgery, The Second Hospital of Dalian Medical University, Dalian, China; 2 Department of General Surgery, Beijing Huimin Hospital, Beijing, China; 3 Department of General Surgery, Huangyuan County People’s Hospital, Xining, China; 4 Department of Clinical Laboratory, The Second Hospital of Dalian Medical University, Dalian, China

**Keywords:** acute mesenteric ischemia, death, influencing factors, nomogram, predictive model

## Abstract

**Objectives:**

Acute mesenteric ischemia (AMI) is a rare disease with a relatively high mortality rate. We aimed to investigate the factors influencing in-hospital mortality in AMI patients and develop a nomogram for early risk stratification.

**Methods:**

Clinical data of AMI patients hospitalized from January 2013 to October 2025 were retrospectively analyzed. Univariate and multivariate logistic regression identified independent pre-treatment risk factors, and a nomogram was constructed. Discrimination, calibration, and clinical utility were evaluated using receiver operating characteristic curve analysis, Hosmer-Lemeshow test, calibration curves, and decision curve analysis (DCA), with Bootstrap internal validation. Subgroup validation was performed by age, sex, and other subgroups. A further adjusted multivariate model examined associations of early clinical characteristics and treatments with mortality.

**Results:**

Among 235 patients, 44 died in hospital (18.7%). Advanced age, heart failure, higher white blood cell (WBC), and creatinine were independent predictors. The model showed good discrimination (Bootstrap area under the curve: 0.849, 95% CI: 0.780–0.909) and fit (Hosmer-Lemeshow: *χ*² = 10.111, *P* = 0.257), with good calibration and net benefit on DCA. Performance remained stable across subgroups. Further analysis that incorporated early clinical characteristics and treatment measures found that age, heart failure, WBC, aspartate aminotransferase, anticoagulation therapy, and vasopressor use were associated with mortality. Anticoagulation therapy was negatively correlated with mortality risk, whereas vasopressor use was positively correlated with mortality risk.

**Conclusions:**

This study identified risk factors for in-hospital mortality in AMI patients and developed a predictive nomogram model. This aids in early high-risk patient identification, timely intervention, optimized treatment decisions, and improved patient outcomes.

## 1 Introduction

Acute mesenteric ischemia (AMI) is a rare but life-threatening condition caused by insufficient intestinal blood supply. A multicenter prospective observational study reported an occurrence rate of only 0.038% among emergency care hospitals in a global cohort (1). Although the overall occurrence rate of AMI is low, its clinical course progresses rapidly, with a very narrow diagnostic and therapeutic window. When ischemia extends to the muscular and serosal layers (approximately 12 h), irreversible bowel necrosis may develop, leading to complications such as intestinal obstruction, diffuse peritonitis, septic shock, and multiple organ dysfunction syndrome (MODS) (2, 3), all of which markedly increase mortality. Recent global multicenter data show in-hospital and 90-day mortality rates as high as 49% and 53.3%, respectively (1).

Currently, early diagnosis and management of AMI remain challenging. First, the clinical presentation of most AMI patients is non-specific and can easily be confused with other causes of acute abdominal pain (4). Second, meta-analyses indicate that no single biomarker provides sufficient diagnostic accuracy for AMI (5, 6). Computed tomography angiography (CTA) has become the preferred diagnostic modality, with reported sensitivity and specificity of 92.0% and 98.8%, respectively, yet non-vascular imaging features alone do not reliably differentiate AMI subtypes or predict transmural necrosis (7). Furthermore, the treatment options for AMI are advancing rapidly, but recent evidence indicates that both treatment choices and their efficacy are significantly influenced by disease severity (8). Consequently, effective AMI management depends on early identification and risk stratification using limited initial clinical data to enable early prognostic assessment and mortality prediction. It remains critically important to clarify the independent associations between early clinical features, therapeutic interventions, and mortality outcomes after controlling for potential confounders like disease severity, as this can inform optimized individualized treatment and improve physician-patient communication.

In recent years, several studies have examined predictors of mortality or poor outcomes in AMI. Otto et al. reported that postoperative mortality was associated with white blood cell (WBC) count, lactate, bilirubin, creatinine (CREA), etiology, and portomesenteric venous gas (9). A French retrospective study further suggested that early radical anticoagulation may independently reduce mortality, whereas an admission lactate level >3.31 mmol/L independently predicted one-month postoperative death (10). Moreover, Wu et al. found that in occlusive AMI, a longer admission-to-surgery interval, lower platelet (PLT) count, and occlusive arterial AMI (OAAMI) were independently associated with 30-day mortality after exploratory laparotomy (11).

Given the complex etiology, relative rarity, and marked heterogeneity of AMI, prior studies vary substantially in design, endpoint definitions, and reporting, precluding a consistent set of prognostic risk factors. Consequently, accurate assessment of in-hospital mortality risk in AMI remains challenging. In this study, we retrospectively collected clinical data from AMI inpatients at The Second Hospital of Dalian Medical University and stratified patients by clinical outcomes. We conducted a two-stage analysis: first, we developed and internally validated an early mortality risk prediction model using pre-treatment clinical variables and translated it into a visual tool for rapid bedside assessment; second, after accounting for early clinical characteristics, we examined the associations between therapeutic interventions and mortality outcomes to inform optimization of diagnostic and treatment pathways and ultimately improve prognosis.

## 2 Materials and methods

### 2.1 Study population

After receiving approval from the Ethics Committee of The Second Hospital of Dalian Medical University (Number: KY2025-747-01), we conducted a retrospective analysis of clinical data from AMI patients admitted between January 2013 and October 2025. A total of 235 patients meeting the specified criteria were included in the study. Inclusion criteria comprised patients with abdominal pain and a confirmed AMI diagnosis through imaging, surgical exploration, or postoperative pathology indicating thrombi or emboli in mesenteric arteries or veins. Exclusion criteria encompassed: individuals under 18 years old; pregnant patients; those who declined further treatment and left the hospital post-diagnosis; cases of chronic mesenteric ischemia; non-occlusive mesenteric ischemia (NOMI); intestinal ischemia from alternate causes (e.g., intestinal volvulus, intussusception, trauma); severe concurrent infectious diseases; advanced malignant tumors; and patients with significant incomplete clinical data.

### 2.2 Clinical data

Clinical data collected from patients included age, gender, clinical manifestations, comorbidities, history of abdominal surgery, etiology, imaging findings, laboratory test results, treatment strategies, and clinical outcomes. All imaging and laboratory data represent findings from emergency or initial post-admission examinations conducted prior to treatment initiation. Based on collected results, the neutrophil-to-lymphocyte ratio (NLR) and platelet-to-lymphocyte ratio (PLR) were calculated.

Based on treatment strategies during hospitalization, patients undergoing endovascular intervention in addition to medical therapy, and not receiving open revascularization or surgical exploration/resection during hospitalization, were defined as the endovascular therapy group. Patients undergoing laparoscopic or exploratory laparotomy to assess intestinal blood supply in addition to medical therapy, with corresponding surgical interventions based on intraoperative findings, were classified into the surgical therapy group. Combined treatment involves receiving both revascularization and surgery during hospitalization, including additional surgery following endovascular treatment due to the presence (or progression) of intestinal ischemia, as well as further open revascularization and resection of necrotic bowel segments after exploratory laparotomy.

All treatment plans were developed through multidisciplinary team (MDT) discussions and implemented based on informed consent following shared decision-making with patients and their families.

### 2.3 Data preprocessing

The collected clinical data underwent preprocessing, during which imaging and laboratory findings were systematically retrieved and verified. Variables with ≥ 10% missing data were excluded, while the remaining variables were addressed for missing values through multiple interpolation to mitigate the impact of missing data on the results.

### 2.4 Statistical analysis

Continuous variables are reported as mean ± standard deviation for normally distributed data and as median and interquartile range, M [P25, P75], for non-normally distributed data. Between-group comparisons are conducted using appropriate methods such as independent-samples *t* test or Mann–Whitney *U*-test. Categorical variables are presented as frequencies and percentages [*n* (%)], and comparisons are performed using the *χ*² test or Fisher's exact test. Variables demonstrating significant between-group differences are included in a collinearity assessment, and the variance inflation factor (VIF) is computed. Variables with VIF >5 are eliminated to mitigate multicollinearity. The remaining variables are included in a multiple logistic regression model (forward: LR) to identify independent factors linked to in-hospital death from AMI, with odds ratios (ORs) and 95% confidence intervals (CIs) reported.

A logistic regression equation was derived from multivariate analysis to predict in-hospital mortality risk for AMI, visualized using a nomogram. Discriminative ability was assessed with the receiver operating characteristic (ROC) curve, and the area under the curve (AUC) was calculated. The DeLong test compared AUCs between the overall model and single indicators. Internal validation was conducted with 1,000 bootstrap resamples. Model fit was evaluated using the Hosmer-Lemeshow test. A calibration curve assessed agreement between predicted and observed probabilities, and decision curve analysis (DCA) evaluated clinical benefit. Model performance across AMI subgroups was assessed by stratifying patients based on age, sex, comorbidity status, and etiology. AUC, sensitivity, and specificity were calculated for each subgroup, and the DeLong test compared AUCs within each classification.

In addition, to further evaluate the correlation between treatment measures and the prognosis of AMI, univariate and multivariate logistic regression (forward: LR) were performed based on early clinical characteristics and treatment measures.

Statistical analyses utilized SPSS 27.0 and R 4.4.1, employing two-tailed tests with a significance threshold of *P* < 0.05.

## 3 Results

### 3.1 Comparison of pre-treatment clinical data between the two groups of patients

Among the 235 patients with AMI included in this study, 191 survived and 44 died, with an overall mortality rate of 18.7%. The patients' clinical characteristics are detailed in Table 1. Overall, the age of AMI patients was 68.00 [58.00, 76.00] years. Notably, the deceased patients were older compared to the survivors, with a statistically significant age gap between the two groups (77 vs. 67, *P* < 0.001). Gender distribution did not exhibit a significant difference between the cohorts.

**Table 1:** Comparison of pre-treatment clinical data between the survival group and the death group.

Variable	Overall (*n* = 235)	Survival (*n* = 191)	Non-survival (*n* = 44)	Statistical value	*P*
Age (years)	68.00 [58.00, 76.00]	67.00 [55.00, 74.00]	77.00 [69.00, 86.00]	*Z* = −5.413	<0.001
Gender, *n* (%)				*χ*² = 1.482	0.223
Female	88 (37.4%)	68 (35.6%)	20 (45.5%)		
Male	147 (62.6%)	123 (64.4%)	24 (54.5%)		
Abdominal pain, *n* (%)	230 (97.9%)	186 (97.4%)	44 (100.0%)	–	0.587
Abdominal distension, *n* (%)	91 (38.7%)	79 (41.4%)	12 (27.3%)	χ² = 2.991	0.084
Diarrhea, *n* (%)	26 (11.1%)	20 (10.5%)	6 (13.6%)	–	0.594
Nausea and vomiting, *n* (%)	98 (41.7%)	79 (41.4%)	19 (43.2%)	χ² = 0.049	0.825
Decreased or absent flatulence and bowel movements, *n* (%)	40 (17.0%)	31 (16.2%)	9 (20.5%)	χ² = 0.452	0.501
Gastrointestinal bleeding, *n* (%)	33 (14.0%)	30 (15.7%)	3 (6.8%)	χ² = 2.341	0.126
Peritonitis, *n* (%)	57 (24.3%)	45 (23.6%)	12 (27.3%)	χ² = 0.268	0.604
Diabetes, *n* (%)	66 (28.1%)	47 (24.6%)	19 (43.2%)	χ² = 6.109	0.013
Coronary heart disease, *n* (%)	41 (17.4%)	28 (14.7%)	13 (29.5%)	χ² = 5.502	0.019
Atrial fibrillation, *n* (%)	98 (41.7%)	69 (36.1%)	29 (65.9%)	χ² = 13.048	<0.001
Heart failure, *n* (%)	17 (7.2%)	6 (3.1%)	11 (25.0%)	–	<0.001
CKD, *n* (%)	9 (3.8%)	5 (2.6%)	4 (9.1%)	–	0.066
Liver disease, *n* (%)	22 (9.4%)	19 (9.9%)	3 (6.8%)	–	0.774
History of abdominal surgery, *n* (%)	65 (27.7%)	54 (28.3%)	11 (25.0%)	χ² = 0.191	0.662
Etiology, *n* (%)				–	<0.001
Venous	100 (42.6%)	94 (49.2%)	6 (13.6%)		
Arterial	126 (53.6%)	90 (47.1%)	36 (81.8%)		
Venous and arterial	9 (3.8%)	7 (3.7%)	2 (4.5%)		
Intestinal dilatation, *n* (%)	77 (32.8%)	57 (29.8%)	20 (45.5%)	χ² = 3.956	0.047
Intestinal wall thickening, *n* (%)	84 (35.7%)	70 (36.6%)	14 (31.8%)	χ² = 0.363	0.547
Ascites, *n* (%)	100 (42.6%)	85 (44.5%)	15 (34.1%)	χ² = 1.586	0.208
WBC (*10^9^/L)	12.41 [8.85, 17.33]	11.73 [8.25, 15.81]	17.07 [12.27, 21.84]	*Z* = −4.158	<0.001
NLR	10.65 [4.95, 20.76]	9.63 [4.48, 18.59]	17.82 [9.02, 30.16]	*Z* = −3.852	<0.001
PLR	204.21 [117.61, 326.53]	182.89 [111.60, 298.65]	241.50 [164.27, 400.36]	*Z* = −2.332	0.020
PLT (*10^9^/L)	204.00 [160.00, 265.00]	203.00 [154.00, 265.00]	207.00 [166.25, 263.50]	*Z* = −0.566	0.572
Fbg (g/L)	4.67 [3.54, 5.98]	4.56 [3.47, 5.72]	5.17 [3.63, 7.16]	*Z* = −1.925	0.054
D-dimer (μg/mL)	2.55 [1.48, 5.81]	2.60 [1.32, 6.26]	2.41 [1.70, 4.38]	*Z* = −0.034	0.973
ALT (U/L)	26.90 [18.70, 42.00]	26.80 [18.60, 41.30]	27.42 [18.93, 44.60]	*Z* = −0.299	0.765
AST (U/L)	29.00 [20.60, 40.00]	27.40 [20.30, 38.20]	34.70 [26.01, 48.78]	*Z* = −3.043	0.002
ALB (g/L)	38.70 [34.40, 43.30]	38.90 [34.24, 43.09]	38.55 [36.38, 44.93]	*Z* = −1.146	0.252
CREA (μmol/L)	78.20 [59.40, 97.50]	74.00 [57.80, 91.00]	108.86 [84.67, 210.43]	*Z* = −5.108	<0.001

The “–” in the statistical value indicates the use of Fisher's exact test. CKD, chronic kidney disease; WBC, white blood cells; NLR, neutrophil-to-lymphocyte ratio; PLR, platelet-to-lymphocyte ratio; PLT, platelet; Fbg, fibrinogen; ALT, alanine aminotransferase; AST, aspartate transaminase; ALB, albumin; CREA, creatinine.

Additionally, we observed higher proportions of patients with underlying conditions such as diabetes (43.2% vs. 24.6%, *P* = 0.013), coronary heart disease (29.5% vs. 14.7%, *P* = 0.019), atrial fibrillation (65.9% vs. 36.1%, *P* < 0.001), and heart failure (25.0% vs. 3.1%, *P* < 0.001) in the deceased group compared to the surviving group. There were also notable disparities in the etiologies of AMI between the surviving and deceased groups (*P* < 0.001). Specifically, the prevalence of OAAMI was substantially higher in the deceased group (81.8%) than in the surviving group (47.1%), whereas the incidence of MVT patients in the surviving group (49.2%) was markedly higher than in the deceased group (13.6%). Imaging and laboratory findings indicated a higher likelihood of intestinal dilation and elevated levels of WBC, NLR, PLR, aspartate transaminase (AST), and CREA in the deceased group. Conversely, there were no significant variations between the two groups in terms of clinical manifestations, physical examinations, chronic kidney disease (CKD), liver disease, history of abdominal surgeries, imaging results of intestinal wall thickening and ascites changes, PLT, fibrinogen (Fbg), D-dimer, alanine aminotransferase (ALT), and albumin (ALB).

### 3.2 Identify the key predictive factors for in-hospital mortality in patients with AMI

We performed a collinearity test on indicators that showed statistical significance in the differential analysis between the two patient groups. All VIFs were less than 5, indicating no multicollinearity (Supplementary Table S1). Therefore, we included all variables in the multivariate analysis (Forward: LR). The analysis showed that age (OR: 1.093, 95% CI: 1.048–1.140, *P* < 0.001), heart failure (OR: 7.507, 95% CI: 2.192–25.707, *P* = 0.001), WBC (OR: 1.086, 95% CI: 1.025–1.150, *P* = 0.005), and CREA (OR: 1.006, 95% CI: 1.003–1.009, *P* < 0.001) were independent risk factors for death in patients with AMI (Table 2 and Supplementary Figure S1).

**Table 2:** Multivariate logistic regression analysis.

Variable	*β*	SE	Wald	*P*	OR	95%CI	
						Lower	Upper
Age (years)	0.089	0.021	17.326	<0.001	1.093	1.048	1.140
Heart failure	2.016	0.628	10.304	0.001	7.507	2.192	25.707
WBC (*10^9^/L)	0.082	0.029	7.926	0.005	1.086	1.025	1.150
CREA (μmol/L)	0.006	0.002	15.511	<0.001	1.006	1.003	1.009
Constant	−10.066	1.766	32.504	<0.001	0.000		

OR, odds ratio; CI, confidence interval; WBC, white blood cell; CREA, creatinine.

### 3.3 Development and evaluation of a clinical predictive model for in-hospital death in AMI patients

**Figure 1:**
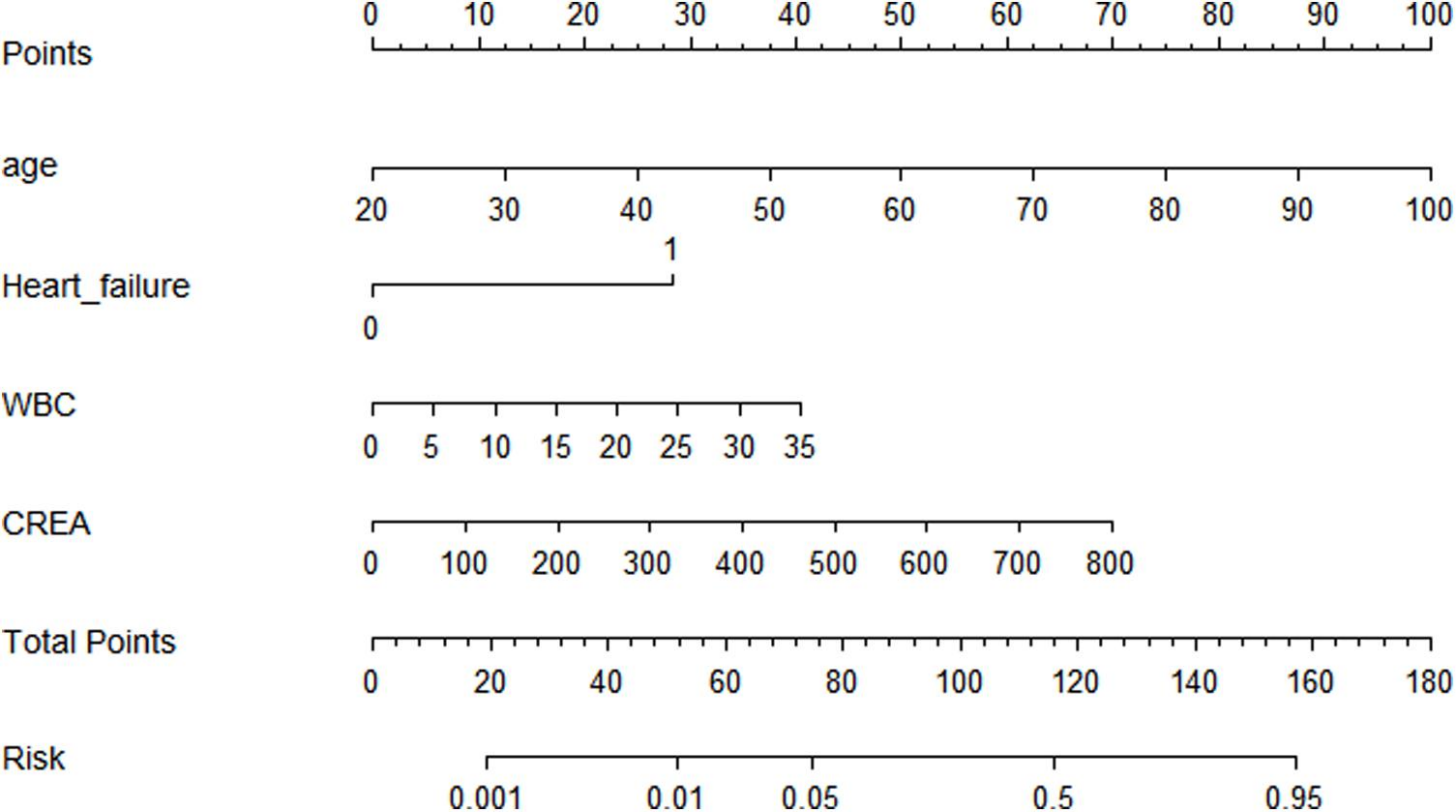
Nomogram model for predicting in-hospital mortality in AMI patients.

Based on the four independent risk factors screened by multivariate logistic regression, a prediction model for in-hospital death risk of AMI was constructed, and the following logistic regression equation was obtained: logit(P) = −10.066 + 0.089 * age +2.016 * accompanied by heart failure +0.082 * WBC +0.006 * CREA, which was visually presented in the form of a nomogram (Figure 1). In clinical application, the total score can be obtained by accumulating the scores corresponding to each risk factor through the nomogram, so as to predict the matching in-hospital death risk. A higher total score indicates a greater in-hospital death risk.

**Figure 2:**
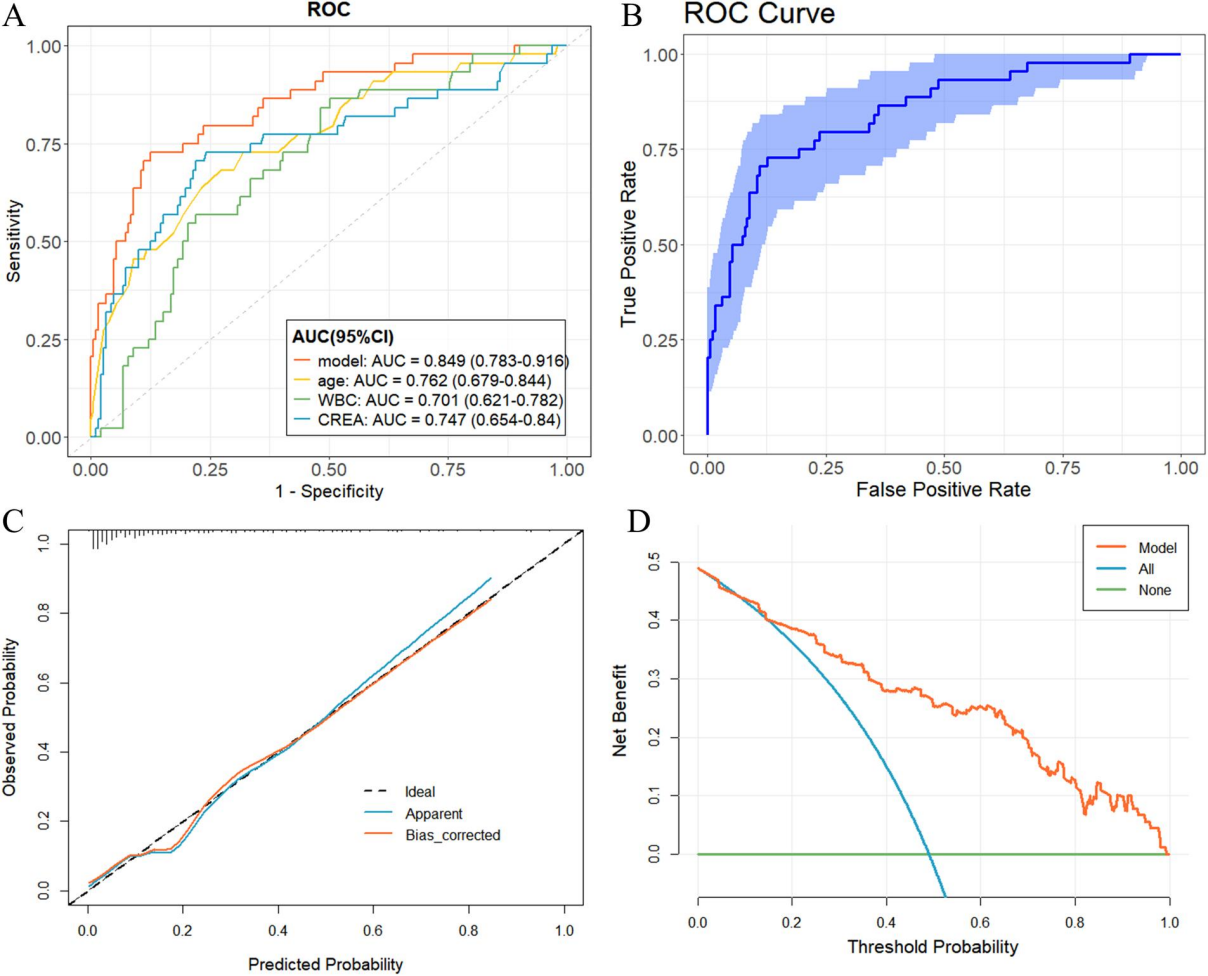
Evaluation and validation of the nomogram model. **(A)** Comparison of receiver operating characteristic (ROC) curves of the prediction model and individual predictors. **(B)** Bootstrap ROC curve of the prediction model. **(C)** Calibration curve. **(D)** Decision curve analysis.

By plotting ROC curves for the prediction model and for age, WBC, and CREA (Figure 2A), we found that the model had the highest AUC, 0.849 (95% CI: 0.783–0.916). The DeLong test further showed that the model combining the four variables had better discriminative efficacy than any single indicator, and the difference was statistically significant (Supplementary Table S2). After 1,000 repeated Bootstrap samples for internal validation, the model AUC remained 0.849 (95% CI: 0.780–0.909), indicating that its discriminatory ability was stable and reliable (Figure 2B). The Hosmer-Lemeshow test indicated adequate fit (*χ*² = 10.111, *P* = 0.257). The calibration curve (Figure 2C) showed close agreement between predicted probabilities and observed event rates, confirming excellent calibration. DCA (Figure 2D) indicated that across a wide range of threshold probabilities (0.09–0.99), the nomogram provided higher net benefit than either the “treat-all” or “treat-none” strategies, suggesting potential clinical value for risk stratification and decision support.

### 3.4 Subgroup analysis

**Figure 3:**
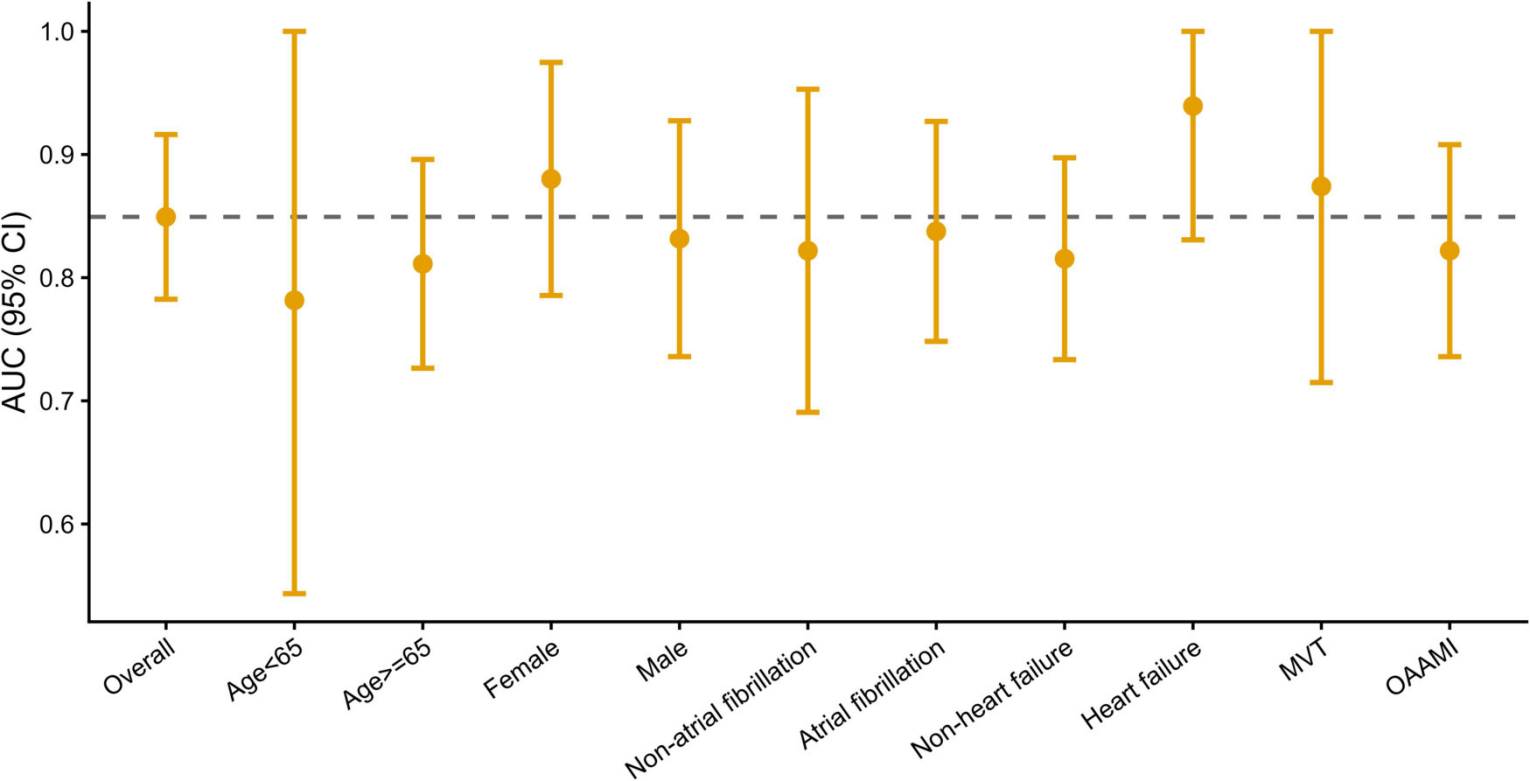
Comparison of AUC among different clinical subgroups.

To evaluate the robustness of the prediction model under different clinical conditions, we further conducted subgroup analyses according to age, gender, presence of atrial fibrillation, presence of heart failure, and etiology type (Figure 3 and Table 3). The results showed that the model maintained a stable discriminatory ability in each stratified population, with the AUC of subgroups ranging from 0.782 to 0.939, suggesting that the model had relatively consistent discriminatory performance in populations with different clinical characteristics. The AUC of some subgroups showed a relatively high trend. For example, the AUC of the subgroup with heart failure was 0.939 (95% CI: 0.831–1.000), and its specificity was 1.000 (95% CI: 0.541–1.000). In addition, the AUCs of the female subgroup and the MVT subgroup were also relatively high, which were 0.880 (95% CI: 0.786–0.975) and 0.874 (95% CI: 0.715–1.000), respectively. In terms of sensitivity and specificity, the sensitivity of each subgroup ranged from 0.636 to 0.833, and the specificity ranged from 0.713 to 1.000.

**Table 3:** Comparison of performance of different subgroups of the prediction model.

Subgroup	AUC (95% CI)	Sensitivity (95% CI)	Specificity (95% CI)	*P* (DeLong's test)
Overall	0.849 (0.783, 0.916)	0.727 (0.572, 0.850)	0.874 (0.819, 0.918)	–
Age<65	0.782 (0.543, 1.000)	0.833 (0.359, 0.996)	0.713 (0.606, 0.805)	0.819
Age≥65	0.811 (0.726, 0.896)	0.737 (0.569, 0.866)	0.817 (0.729, 0.886)	
Female	0.880 (0.786, 0.975)	0.800 (0.563, 0.943)	0.897 (0.799, 0.958)	0.481
Male	0.832 (0.736, 0.927)	0.667 (0.447, 0.844)	0.902 (0.836, 0.949)	
Non-atrial fibrillation	0.822 (0.691, 0.953)	0.667 (0.384, 0.882)	0.918 (0.854, 0.960)	0.846
Atrial fibrillation	0.838 (0.748, 0.927)	0.759 (0.565, 0.897)	0.855 (0.750, 0.928)	
Non-heart failure	0.815 (0.733, 0.897)	0.636 (0.451, 0.796)	0.892 (0.838, 0.933)	0.082
Heart failure	0.939 (0.831, 1.000)	0.818 (0.482, 0.977)	1.000 (0.541, 1.000)	
MVT	0.874 (0.715, 1.000)	0.667 (0.223, 0.957)	0.957 (0.895, 0.988)	0.573
OAAMI	0.822 (0.736, 0.908)	0.778 (0.608, 0.899)	0.822 (0.727, 0.895)	

To compare the differences in discriminative ability among different levels of the same stratification factor, we used the DeLong test to conduct pairwise comparisons of the AUCs between two subgroups within each stratification factor. The results showed that all *P* values of the relevant comparisons were >0.05 (Table 3), suggesting that there were no statistically significant differences in the AUCs among different levels within each stratification factor. In summary, the model demonstrated consistent discriminative performance among the populations stratified by age, gender, comorbidities, and etiology, indicating good stability and generalization ability.

### 3.5 Comparison of treatment methods between the two groups of patients

After constructing and validating a mortality risk prediction model for AMI patients based on early clinical characteristics, we further compared the differences in major treatment measures during hospitalization between the two groups of patients (Table 4) to assess the influencing factors of mortality in AMI patients after treatment.

**Table 4:** Comparison of treatment measures between the survival group and the death group.

Variable	Overall (*n* = 235)	Survival (*n* = 191)	Non-survival (*n* = 44)	Statistical value	*P*
Antiplatelet therapy, *n* (%)	42 (17.9%)	31 (16.2%)	11 (25.0%)	χ² = 1.874	0.171
Anticoagulant therapy, *n* (%)	224 (95.3%)	185 (96.9%)	39 (88.6%)	–	0.035
Thrombolytic therapy, *n* (%)	71 (30.2%)	54 (28.3%)	17 (38.6%)	χ² = 1.822	0.177
Vasodilator therapy, *n* (%)	125 (53.2%)	101 (52.9%)	24 (54.5%)	χ² = 0.040	0.842
Vasopressor therapy, *n* (%)	63 (26.8%)	28 (14.7%)	35 (79.5%)	χ² = 76.733	<0.001
Surgical/Interventional Procedures, *n* (%)	159 (67.7%)	124 (64.9%)	35 (79.5%)	χ² = 3.495	0.062
Endovascular Therapy, *n* (%)	78/159 (49.1%)	59/124 (47.6%)	19/35 (54.3%)	χ² = 0.491	0.483
Surgical treatment, *n* (%)	70/159 (44.0%)	59/124 (47.6%)	11/35 (31.4%)	χ² = 2.890	0.089
Combination therapy, *n* (%)	11/159 (6.9%)	6/124 (4.8%)	5/35 (14.3%)	–	0.065
Gastrointestinal Exploration, *n* (%)	81/159 (50.9%)	65/124 (52.4%)	16/35 (45.7%)	χ² = 0.491	0.483
Extent of intestinal resection, *n* (%)				–	0.180
No need for resection	3/81 (3.7%)	3/65 (4.6%)	0/16 (0.0%)		
Small bowel resection	60/81 (74.1%)	50/65 (76.9%)	10/16 (62.5%)		
Colon resection	2/81 (2.5%)	2/65 (3.1%)	0/16 (0.0%)		
Small intestine and colon resection	15/81 (18.5%)	10/65 (15.4%)	5/16 (31.3%)		
Lethal palliative care (switch-off procedure)	1/81 (1.2%)	0/65 (0.0%)	1/16 (6.3%)		
Intestinal anastomosis, *n* (%)	70/77 (90.9%)	57/62 (91.9%)	13/15 (86.7%)	–	0.617

The “–” in the statistical value indicates the use of Fisher's exact test.

The comparison revealed that patients in the death group were more likely to require maintenance vasopressors (79.5% vs. 14.7%, *P* < 0.001) and had insufficient anticoagulation therapy (88.6% vs. 96.9%, *P* = 0.035). However, there were no notable variances in the utilization of antiplatelet medications, thrombolytics, and vasodilators between the groups.

Moreover, 159 patients (67.7%) underwent surgical intervention in addition to medical therapy. The difference in intervention rates between the mortality and survival groups was not statistically significant (79.5% vs. 64.9%, *P* = 0.062). Of those receiving interventions, 78 (49.1%) had endovascular therapy, 70 (44.0%) underwent surgery, and 11 (6.9%) had combined revascularization and surgery. The proportion of patients with combined therapy was higher in the mortality group (14.3% vs. 4.8%), but the difference was not statistically significant (*P* = 0.065). This trend likely reflects the mortality group's more severe condition and higher comorbidity burden, leading to a preference for aggressive or complex treatments.

In this study, 81 patients underwent gastrointestinal exploration. Three cases (3.7%) showed intestinal ischemia without necrosis and did not undergo resection, 60 cases (74.1%) underwent small intestine resection, 2 cases (2.5%) underwent colon resection, and 15 cases (18.5%) underwent simultaneous resection of both small intestine and colon. One individual manifested extensive ischemia and necrosis throughout the entire small intestine and colon during exploration, leading to palliative intervention following comprehensive consultation with family members. Among patients who underwent intestinal resection, 70 cases (90.9%) received primary intestinal anastomosis. No substantial disparities were noted among groups in either the extent of intestinal resection or the frequency of primary anastomosis.

Significant differences (*P* < 0.05) in pre-treatment clinical characteristics and treatment measures between the survival and death groups were identified. Variables meeting the criteria (VIF < 5, no multicollinearity, as shown in Supplementary Table S3) were included in multivariate logistic regression analysis (Forward: LR). The results are shown in Table 5. Age, comorbid heart failure, WBC, AST levels, anticoagulant therapy, and use of vasopressors were correlated with the death outcome. Among them, anticoagulant therapy was negatively associated with death (OR: 0.079, 95%CI: 0.014–0.454, *P* = 0.004), and the use of vasopressors was positively associated with death (OR: 32.719, 95%CI: 10.363–103.303, *P* < 0.001).

**Table 5:** Multivariate logistic regression analysis integrating early clinical characteristics and treatment measures.

Variable	*β*	SE	Wald	*P*	OR	95%CI	
						Lower	Upper
Age (years)	0.076	0.023	11.094	<0.001	1.079	1.032	1.128
Heart failure	1.994	0.830	5.777	0.016	7.344	1.445	37.328
WBC (*10^9^/L)	0.091	0.037	5.945	0.015	1.095	1.018	1.178
AST (U/L)	0.008	0.003	7.190	0.007	1.008	1.002	1.014
Anticoagulant therapy	−2.541	0.893	8.098	0.004	0.079	0.014	0.454
Vasopressor therapy	3.488	0.587	35.356	<0.001	32.719	10.363	103.303
Constant	−8.255	2.072	15.874	<0.001	0.000		

OR, odds ratio; CI, confidence interval; WBC, white blood cell; AST, aspartate transaminase.

## 4 Discussion

This single-center retrospective study included 235 patients with AMI admitted between January 2013 and October 2025, and the overall in-hospital mortality rate was 18.7%. Compared with the short-term mortality rates (in-hospital/28-day/30-day mortality rates) reported in previous studies (31.0%–55.9%) (1, 9, 12, 13), the mortality rate observed in this study was relatively low, suggesting that the overall prognosis of AMI patients at our center may be improved. This may also be related to differences in the composition of the enrolled population, treatment procedures, and intervention strategies.

Baseline characteristics showed that patients in the death group were notably older than those in the survival group. Multivariate logistic regression analysis confirmed age as an independent predictor of mortality in AMI patients, consistent with prior research findings (12, 14, 15). Elderly patients often have comorbid underlying diseases, with declined immune defense ability and physiological reserve. They have poorer tolerance to infection, ischemia-reperfusion injury, and multiple organ failure, and their clinical manifestations are less typical, which can easily lead to delays in seeking medical attention and diagnosis, thereby increasing the risk of death (16, 17). However, advanced age does not necessarily mean that the poor outcome is irreversible. Vrba et al. suggested that early diagnosis and endovascular revascularization and surgical treatment within 24 h for elderly patients could achieve better efficacy (18). Therefore, elderly patients presenting with abdominal pain should be closely monitored for AMI. Prompt initiation of CTA and MDT assessment is crucial for timely diagnosis. For elderly patients with AMI and underlying conditions, intensive care unit monitoring and aggressive resuscitation and organ support are recommended.

In this study, most patients had comorbid underlying diseases, but only heart failure was independently associated with in-hospital mortality, which was consistent with previous research reports (15, 19). From a pathophysiological perspective, heart failure can lead to a decrease in cardiac output and insufficient effective circulating blood volume, thus causing spasm of the mesenteric blood vessels that are highly sensitive to perfusion changes. Meanwhile, to maintain the perfusion of vital organs such as the heart and brain, the body often constricts the visceral blood vessels through neurohumoral regulation and redistributes blood flow, further reducing mesenteric perfusion (20, 21). On this basis, ischemia and hypoxia of the intestinal wall are aggravated, and if not corrected in time, it is prone to progress to irreversible intestinal necrosis. After the destruction of the intestinal mucosal barrier, bacteria, endotoxins, and inflammatory mediators enter the bloodstream, which can also further aggravate cardiac insufficiency by damaging the myocardium, thus forming a vicious cycle (20, 21). At the same time, patients with heart failure are less tolerant of volume resuscitation, surgical anesthesia, etc. Therefore, for AMI patients with comorbid heart failure, treatment strategies need to emphasize more refined hemodynamic management, and at the same time, actively conduct anti-heart failure treatment.

WBC count is a commonly used peripheral blood indicator reflecting the body's inflammatory response and immune-related status. Previous studies have confirmed its value in the risk stratification and prognosis assessment of AMI (22, 23). The results of this study are consistent with previous evidence. Compared with the survival group, the WBC level was significantly higher in the death group. In the multivariate analysis of early clinical characteristics, after adjusting for potential confounding factors, for every 1 × 10^9^/L increase in WBC, the in-hospital death risk increased by 8.6% (*P* = 0.005), suggesting that an elevated WBC is independently associated with adverse outcomes. Previous studies have also shown that an elevated WBC is associated with the occurrence of intestinal necrosis and an increased need for intestinal resection (24, 25), which may partially explain the link between an elevated WBC and an increased risk of death. Therefore, in clinical practice, the initial level and dynamic changes of WBC should be emphasized to identify high-risk patients early and optimize treatment strategies in a timely manner.

In a multivariate analysis based on pre-treatment clinical characteristics, this study found that elevated CREA was significantly associated with an increased risk of in-hospital mortality. As an important indicator of glomerular filtration function, multiple studies have also supported the association between elevated CREA and decreased survival in AMI (26, 27). Elevated CREA not only reflects the burden of previous CKD but may also indicate acute kidney injury during the progression of AMI. Both can lead to metabolic disorders, accumulation of uremic toxins, imbalance of water, electrolytes and acid-base balance, and promote the release of pro-inflammatory cytokines, thus driving the progression of MODS (28). In addition, impaired renal function may also limit the selection and dosage adjustment of some potentially nephrotoxic drugs (such as some antibacterial drugs), thereby affecting the implementation of comprehensive treatment strategies and creating a vicious cycle. Based on the above considerations, AMI patients with elevated CREA, regardless of whether they have a history of CKD, should be given high-risk assessment and careful management.

Regarding liver function-related indicators, this study observed a significant difference in AST rather than ALT between survivors and non-survivors. Considering that ALT is mainly distributed in the cytoplasm, while AST is not only present in the cytoplasm but also located in the mitochondria of hepatocytes, it suggests that liver function impairment may be more severe in AMI patients who died, involving the mitochondria and leading to a significant increase in AST. Meanwhile, impaired liver function can also reduce its clearance rate, resulting in a further increase in AST and possibly accompanied by an elevated AST/ALT ratio (29). Additionally, AST is also expressed in various organs such as the heart, skeletal muscles, kidneys, and brain tissue. Therefore, its elevation can reflect systemic tissue damage or multi-organ involvement (30, 31). The results of this study are similar to those of Huang et al., who also found that elevated AST levels were associated with a worse prognosis in AMI patients (32). Similarly, a Swiss study also found that among patients undergoing abdominal CT for suspected AMI, those with elevated AST had a significantly increased short-term death risk, with a short-term mortality rate approximately three times that of those with normal AST (33). Based on the existing evidence, AST may be an important clue reflecting the severity of AMI and multi-organ function impairment. It is recommended to strengthen the monitoring of AST levels in clinical management and further clarify its prognostic predictive value in the treatment pathway in subsequent studies.

Previous studies generally show that early anticoagulant therapy reduces mortality risk in patients with AMI (34–38). Our study found that 95% of the 224 patients received anticoagulant therapy, with a notably higher usage rate in survivors compared to non-survivors. Following adjustments for other variables, anticoagulant therapy was linked to lower in-hospital mortality from AMI. Possible mechanisms include promotion of vascular recanalization, inhibition of thrombus propagation, improvement of tissue perfusion, and mitigation of ischemia-reperfusion injury (39). Lakbar et al. observed a higher 30-day survival rate in patients given early full-dose anticoagulation (34). In our study, early anticoagulation primarily comprised nadroparin, enoxaparin, and other low-molecular-weight heparins, which were transitioned to oral anticoagulants (such as warfarin or rivaroxaban) after clinical stabilization. Selection of the anticoagulant agent and dose adjustments were based on PLT, CREA levels, and bleeding risk. Because this study did not compare efficacy, optimal dosing, or treatment duration among different anticoagulants, these questions require clarification in future prospective studies.

In this study, 26.8% patients received vasopressor therapy due to refractory hypotension persisting after fluid resuscitation. Multivariate regression showed that vasopressor use was independently associated with in-hospital death, consistent with previous reports (38). This finding can be interpreted in two ways. First, vasopressors may further reduce mesenteric perfusion through visceral vasoconstriction, thereby inducing occult NOMI (40). Second, the need for vasopressors often indicates circulatory failure and critical illness, which carry a higher risk of death. Thus, both drug effects and disease severity may jointly drive the observed association. Recent studies have offered alternative perspectives. Early achievement of peak vasopressor dose within 6 h of septic shock onset correlated with improved survival and decreased organ support requirement (41). Conversely, delayed peak dose attainment elevated the risk of arrhythmia and mesenteric ischemia (41). That study also provided preliminary evidence that early combination therapy with multiple low-dose vasopressors may be superior to a stepwise single-drug escalation strategy (41). Therefore, the optimal timing to initiate vasopressors, as well as the ideal dosing and combination regimens for AMI, remain to be determined, their potential to improve patient prognosis may be underestimated.

In our study, no clear statistical association was observed between surgical/interventional interventions (including endovascular treatment, surgical treatment, and combined treatment) and in-hospital death. However, among the deceased patients, the proportion of those who received combined treatment showed an increasing trend (14.3% vs. 4.8%), suggesting that the choice of treatment regimen may be related to the complexity of the condition and organ function impairment. A simple horizontal comparison of treatment methods may be biased, and more attention should be paid to the precise matching of treatment strategies with the patient's pathophysiological status. A meta-analysis found that after adjusting for disease severity, the relative advantage of endovascular treatment was significantly weakened (8), which also confirmed the above view from the side. It should be emphasized that the treatment plans for patients in this study were all formulated after MDT evaluation. A relatively standardized diagnosis and treatment process may reduce its impact on the death outcome, which also indicates the potential value of “process management” in the treatment of AMI. A Finnish study showed that implementing the MDT process could increase the enhanced CT examination rate and revascularization rate of AMI patients, shorten the time from admission to surgical intervention, and reduce the 30-day mortality rate from 51% to 25%. Moreover, the study found that MDT management was an independent protective factor for 30-day mortality (42). France also reported a similar organizational model and achieved similar results (43). The above evidence suggests that MDT can facilitate rapid identification and diagnosis of AMI, early resection of ischemic and necrotic bowel segments, and vascular reconstruction, thereby shortening reperfusion time, preventing progression to multiple organ failure, and improving survival rates. In the future, it is still necessary to further optimize and verify the generalizability of MDT in different medical systems and its real benefits for key outcomes of AMI.

This study conducted a multivariate analysis based on readily available pre-treatment clinical data of AMI patients, identifying advanced age, concomitant heart failure, and elevated WBC and CREA levels as independent risk factors for in-hospital mortality. Based on these factors, a mortality risk prediction model for AMI patients suitable for pre-treatment decision-making was constructed. The model's discriminant power was superior to that of a single clinical indicator, and internal validation using the Bootstrap method showed good stability, with an AUC of 0.849 (95% CI: 0.780–0.909). Furthermore, the model maintained relatively consistent predictive performance across different subgroups, indicating its robustness and potential for generalization. This model can achieve quantitative stratification of mortality risk early in hospital admission, providing objective evidence for rapid identification of high-risk patients, early initiation of MDT approaches, and optimization of treatment processes. Further, by integrating early clinical characteristics and treatment measures, this study found an independent correlation between anticoagulation therapy and vasopressor use and mortality outcomes, providing reference clues for improving early treatment pathways and management strategies for fluid resuscitation and tissue perfusion.

This study still has several limitations. First, this study was a single-center retrospective study with a relatively limited sample size and a low number of deaths, which may affect the robustness of the model. Furthermore, it lacks external validation, and the generalizability of the results requires further confirmation through multicenter prospective studies. Second, the study population was limited to hospitalized patients. Patients who were not hospitalized or who discontinued treatment in the outpatient or emergency department were not included, and hospitalized patients who refused further treatment or left the hospital voluntarily were excluded, which may lead to selection bias. In addition, the time span of this study was long, and advances in diagnosis and treatment technology and management processes during this period may have affected the outcomes, resulting in potential time-related confounding. Third, this study incorporated patients with different AMI etiologies into the same model for analysis. Although the subgroup analysis showed that the predictive model showed relatively satisfactory results in both MVT and OAAMI populations, they may have differences in mechanism, treatment and prognosis. In the future, it is still necessary to expand the sample size, conduct stratified analysis based on etiology, strengthen the exploration of prognostic influencing factors, and develop more targeted predictive models. Fourth, the laboratory and imaging examination results in this study were the first examination results after emergency or admission, but there are potential differences in the interval from symptom onset to examination time between patients. Changes in these examination indicators may be affected by ischemia time. Because this study was retrospective, the exact time intervals were not consistently recorded, thus preventing the inclusion of this variable in reliable quantitative analysis. Future prospective studies are needed to collect clinical data at standardized time points for further evaluation and to explore the impact of dynamic changes in examination results at multiple time points on the prognosis of AMI. Besides, some inflammation and metabolism-related indicators (such as C-reactive protein, procalcitonin, blood lactate, etc.) are missing, making it impossible to analyze and explore their efficacy. Fifth, the end point of this study is in-hospital death, and there is a lack of long-term follow-up information after discharge. Therefore, the predictive value of the model for long-term prognosis still needs further evaluation. Sixth, this study did not adequately quantify the time interval from onset to surgery, or the initiation time, dosage, duration, and combination therapy regimens of anticoagulants and vasopressors, which could affect causal inferences. Considering that residual confounding factors cannot be completely ruled out, the impact of treatment methods on in-hospital mortality in AMI still needs further investigation and confirmation.

## 5 Conclusions

This study analyzed pre-treatment clinical data and identified age, heart failure, WBC, and CREA as independent risk factors for in-hospital mortality in AMI. A predictive model for in-hospital mortality risk was constructed based on these four indicators. The model demonstrated good potential for early risk stratification and has some guiding significance, but further multi-center external validation is needed to clarify its clinical applicability. Multivariate analysis combining pre-treatment clinical characteristics and treatment interventions showed that age, heart failure, WBC, AST, and the use of anticoagulation therapy and vasopressors were independently associated with mortality outcomes. These results suggest that the risk of death in AMI is closely related to inflammatory response, organ dysfunction, treatment methods, and resuscitation strategies.

## Data Availability

The original contributions presented in the study are included in the article/Supplementary Material, further inquiries can be directed to the corresponding author/s.
