# Supplementary material for: Predictive factors for mortality in acute mesenteric ischemia
Source: Front Surg. 2026 Apr 10;13:1805697. doi: 10.3389/fsurg.2026.1805697 (PMC13106151; doi:10.3389/fsurg.2026.1805697)
Supplement: Supplementary file 1 [file Datasheet1.docx]

Supplementary Material

**Supplementary Table 1.** Collinearity detection of indicators with P < 0.05 in the analysis of differences in pre-treatment clinical data

| **Variable** | **Tolerance** | **VIF** |
| --- | --- | --- |
| Age | 0.721 | 1.387 |
| Diabetes | 0.845 | 1.183 |
| Coronary heart disease | 0.852 | 1.173 |
| Atrial fibrillation | 0.574 | 1.741 |
| Heart failure | 0.912 | 1.096 |
| Causes | 0.623 | 1.606 |
| Intestinal distension | 0.946 | 1.057 |
| WBC | 0.493 | 2.027 |
| NLR | 0.213 | 4.704 |
| PLR | 0.376 | 2.658 |
| AST | 0.779 | 1.284 |
| CREA | 0.836 | 1.197 |

**Supplementary Table 2.** Comparison of Predictive Models and Single Variables.

| **Comparison group** | **ΔAUC** | **Z-statistic** | ***P-value*** |
| --- | --- | --- | --- |
| model vs age | 0.088 | 2.545 | 0.011 |
| model vs WBC | 0.148 | 3.273 | 0.001 |
| model vs CREA | 0.102 | 2.275 | 0.023 |

**Supplementary Table 3.** Collinearity detection of variables in integrated pre-treatment clinical data and treatment measures.

| **Variable** | **Tolerance** | **VIF** |
| --- | --- | --- |
| Age | 0.702 | 1.424 |
| Diabetes | 0.836 | 1.196 |
| Coronary heart disease | 0.843 | 1.186 |
| Atrial fibrillation | 0.570 | 1.753 |
| Heart failure | 0.897 | 1.115 |
| Causes | 0.613 | 1.630 |
| Intestinal distension | 0.934 | 1.071 |
| WBC | 0.482 | 2.063 |
| NLR | 0.212 | 4.718 |
| PLR | 0.375 | 2.665 |
| AST | 0.773 | 1.294 |
| CREA | 0.765 | 1.308 |
| Anticoagulant therapy | 0.936 | 1.068 |
| Vasopressor therapy | 0.781 | 1.281 |

**
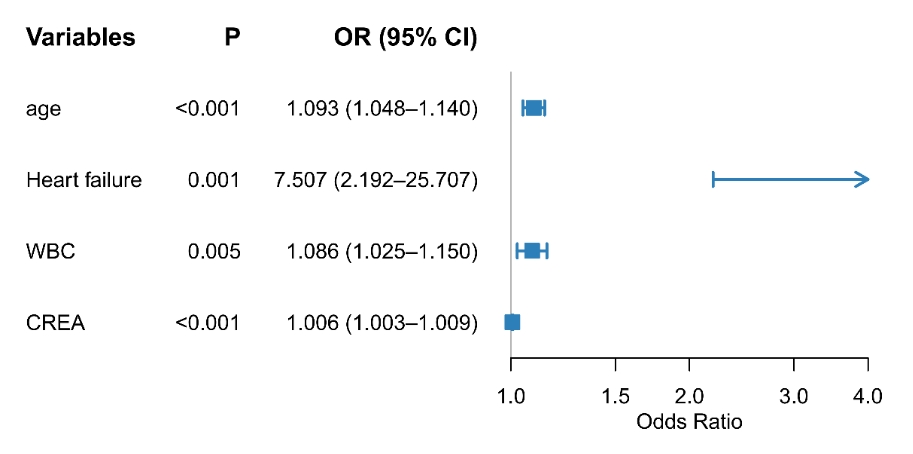
**

**Supplementary Figure 1.** Forest plot of multivariate logistic regression analysis based on pre-treatment clinical data.
